# Correction: Castro et al. Biocompatibility Assessment of Polycaprolactone/Polylactic Acid/Zinc Oxide Nanoparticle Composites Under In Vivo Conditions for Biomedical Applications. *Pharmaceutics* 2023, *15*, 2196

**DOI:** 10.3390/pharmaceutics17091113

**Published:** 2025-08-27

**Authors:** Jorge Iván Castro, Daniela G. Araujo-Rodríguez, Carlos Humberto Valencia-Llano, Diego López Tenorio, Marcela Saavedra, Paula A. Zapata, Carlos David Grande-Tovar

**Affiliations:** 1 Laboratorio SIMERQO, Departamento de Química, Universidad del Valle, Calle 13 No. 100-00, Cali 76001, Colombia; jorge.castro@correounivalle.edu.co; 2 Grupo de Investigación de Fotoquímica y Fotobiología, Universidad del Atlántico, Carrera 30 Número 8-49, Puerto Colombia 081008, Colombia; dgaraujo@mail.uniatlantico.edu.co; 3 Grupo Biomateriales Dentales, Escuela de Odontología, Universidad del Valle, Calle 4B # 36-00, Cali 76001, Colombia; carlos.humberto.valencia@correounivalle.edu.co (C.H.V.-L.); diego.lopez.tenorio@correounivalle.edu.co (D.L.T.); 4 Grupo de Polímeros, Facultad de Química y Biología, Universidad de Santiago de Chile, Santiago 9170020, Chile; alecram.saavedra@gmail.com (M.S.); paula.zapata@usach.cl (P.A.Z.)

## Error in Figure

In the original publication [1], there was a mistake in Figure 6 as published. We mistakenly duplicated a macroscopic photo of a biomodel implanted with different scaffolds, previously published in [2]. This error occurred because we usually use the same animal biomodels to evaluate different materials simultaneously, employing a subdermal implantation model that allows the creation of up to 10 pockets per animal, each containing a different material. In some cases, two or three different systems were implanted in the same biomodel, as per the experimental design. This experimental strategy optimizes animal use, minimizes inter-individual variability, and facilitates direct comparisons between the materials studied under controlled conditions, thereby complying with the principles of the 3Rs (Replacement, Reduction, and Refinement).

The figure in question corresponds to a macroscopic image of the dorsal area of implantation in the biomodel. This type of image is typically included as a preliminary visual representation of the tissue response. It serves as complementary evidence to the histological findings, which constitute the primary support for the article’s conclusions. The figure, intended solely as a preliminary visual context, does not affect the scientific validity, results, or conclusions of the article. The corrected Figure 6 appears below. The authors state that the scientific conclusions are unaffected. This correction was approved by the Academic Editor. The original publication has also been updated.

## Figures and Tables

**Figure 6:**
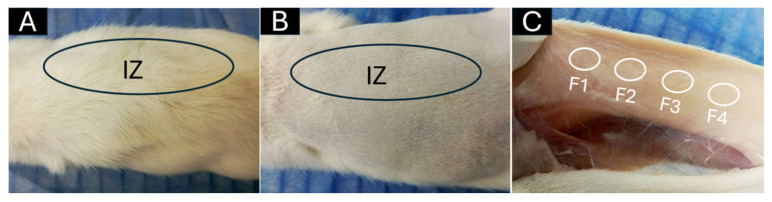
Subdermal dorsal implantation zone. (**A**) Dorsal area with abundant hair. (**B**) Trichotomy of dorsal area. (**C**) Subdermal implantation area. Black ovals: implantation zone. White circles: blocks implanted. F1–F4: formulations 1, 2, 3, and 4. IZ: Implantation zone.
